# Health benefits and application of polysaccharides from *Polygonatum cyrtonema* Hua

**DOI:** 10.3389/fphar.2025.1614596

**Published:** 2025-07-18

**Authors:** Xiaoyan Huang, Xingying Chen, Faming Jiang

**Affiliations:** ^1^ Faculty of Modern Agriculture, Yibin Vocational and Technical College, Yibin, China; ^2^ Department of Traditional Chinese Veterinary Medicine Assessment, Engineering Center of Agricultural Biosafety Assessment and Biotechnology, Yibin Vocational and Technical College, Yibin, China; ^3^ Yibin Sub-Center of Key Laboratory of Sichuan Province for Bamboo Pests Control and Resource Development, Yibin Vocational and Technical College, Yibin, China

**Keywords:** *Polygonatum cyrtonema* Hua, polysaccharides, health benefits, pharmacological mechanisms, medicinal food application

## Abstract

*Polygonatum cyrtonema* Hua is a herbaceous plant renowned for its dietary and medicinal properties. Research has demonstrated that the polysaccharides derived from *P. cyrtonema* Hua exhibit significant anti-inflammatory, antioxidant, immunomodulatory, anti-cancer, anti-fatigue, anti-depression, and anti-obesity activities, as supported by both *in vitro* studies and animal experiments. They also help balance intestinal flora, regulate blood sugar, and prevent cardiovascular disease and Alzheimer’s disease. These effects are mediated through various signaling pathways, such as the mitogen-activated protein kinase, nuclear factor kappa-B, and nuclear factor erythroid 2-related factor 2/heme oxygenase-1 antioxidant signaling. The versatility of polysaccharides from *P. cyrtonema* Hua extends to applications in food and medicine, promoting overall health and wellbeing. This article reviews the pharmacological activities of polysaccharides in *P. cyrtonema* Hua and the factors affecting their content and structure, with particular emphasis on the *Paozhi* processing technique. This review highlights the multifaceted health benefits of these polysaccharides, underscoring their potential as functional food and therapeutic agents. Future research could focus on clinical studies, bioavailability, pharmacokinetics, and structure-function relationship to further validate these health claims and explore the underlying mechanisms of its bioactive compounds.

## 1 Introduction

The genus *Polygonatum* (family Liliaceae) encompasses over 71 species worldwide, distributed across North America, Europe, Russia, India, Korea, Japan, and China (Liu et al., 2023a; Zhang Q. et al., 2024). The majority of them are valued for their significant medicinal and dietary properties (Li, et al., 2023a). In China, *P. rhizoma*, commonly known as *Huangjing*, is a traditional medicinal and edible herb (Zhang Q. et al., 2024). Derived from rhizomes of several *Polygonatum* species, *Huangjing* has been used as an alternative food source since antiquity (Xu et al., 2021). According to the historical texts such as the *Mingyi Bielu*, prolonged consumption of *P. rhizoma* could enhance physical strength, slow the aging process, and alleviate hunger (Xu et al., 2021). Similarly, the *Baopuzi Neipian* records that *P. rhizoma* could be used food during times of famine (Pan et al., 2024).

**FIGURE 1:**
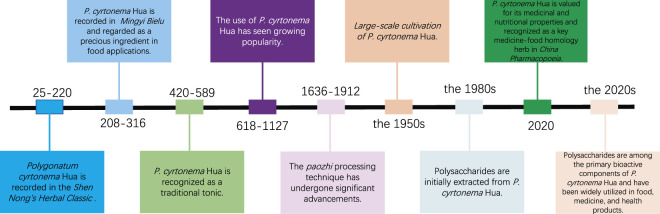
From tradition to innovation: historical and modern applications of *Polygonatum cyrtonema* Hua.

The primary species of *Polygonati rhizoma* include *Polygonatum cyrtonema* Hua (PCH), *Polygonatum sibiricum* Red., and *Polygonatum kingianum* Coll. et Hemsl., distinguished by the appearance of their roots (Chen L. et al., 2022; Shi et al., 2023). Among these, the rhizome of *P. cyrtonema* Hua is most commonly used in both medicine and food (Hu et al., 2022; Luo et al., 2025). PCH is predominantly found in Japan, China, Korea, Pakistan, Afghanistan, and India (Zhao et al., 2018). In China, PCH is highly esteemed for its medicinal and nutritional value and is recognized as a significant medicine-food homology herb in the *China Pharmacopoeia* (2020 edition) (Xie et al., 2023). PCH was initially recorded in the *Shen Nong’s Herbal Classic* (Eastern Han Dynasty, 25–220 AD) (Liu et al., 2024). Historical records reveal that PCH has been used medicinally for about 2000 years, and its major health benefits are derived from the rhizomes (Zhang Y. et al., 2024) (Figure 1).

Traditionally, it is known for promoting salivary fluid secretion, nourishing the lung and Yin, replenishing essence and *qi*, tonifying the spleen and kidney, and strengthening muscles and bones (Lu et al., 2024; Yang G. et al., 2024). Additionally, it protects the cardiovascular system, combats fatigue, and enhances spleen function. It has been used to alleviate dizziness and respiratory illnesses (Zhang Y. et al., 2024). Presently, PCH is included on the “List of Items that Rre Both Food and Medicine (China)” (Xiao et al., 2024). It is rich in various compounds, such as lectins, alkaloids, flavonoids, saponins, and polysaccharides (Pan et al., 2025). Pharmacological research has demonstrated that PCH possesses numerous activities, including immunomodulatory, anti-inflammatory, antioxidant, anti-tumor, anti-bacterial, anti-aging, hypoglycemic and hypolipidemic, and cardiovascular protective effects (Gong H. et al., 2024; Zhang Q. et al., 2024).

The medicinal and dietary properties of PCH are primarily attributed to the bioactive compounds found in its rhizome (Shen et al., 2021). Numerous modern studies have identified that polysaccharides as the principal active compounds in PCH (Zhang et al., 2019). These saccharides contribute significantly to the plant’s therapeutic effects and nutritional value.

With an aging population and increasing emphasis on nutritious and healthy diets, PCH, with its dual use in food and medicine, has attracted considerable interest because of its numerous health advantages. This article reviews the pharmacological activities of polysaccharides in PCH and the factors affecting their content and structure, with particular emphasis on the *Paozhi* processing technique. Additionally, this review explores the underlying mechanism and potential applications of PCH in food, medicine, and cosmetics, aiming to highlight its beneficial chemical profile and properties, and to offer useful references for its development and utilization.

## 2 Materials and methods

Data for this review were collected from 2015 to 2025 using well-known academic databases and platforms, such as PubMed, Web of Science, ScienceDirect, and Google Scholar. Search terms including “*P. cyrtonema* Hua,” “polysaccharides,” “pharmacological activities,” “*Paozhi* processing,” and “medicinal and dietary properties” were used to identify relevant studies. Literature was filtrated according to its relevance to the pharmacological and nutritional properties of *P. cyrtonema* Hua. The collected data were analyzed to provide a comprehensive overview of the plant’s health benefits and applications.

## 3 Polysaccharides in *Polygonatum cyrtonema* Hua

Polysaccharides (16.92%–28.48%) are the primary type of carbohydrates in PCH (Lu et al., 2024). Starch, constituting 7.35%–10.14%, is the predominant polysaccharide. PCH polysaccharide (PCHP) is also rich in dietary fiber, primarily consisting of insoluble hemicellulose, as well as soluble pectin and resistant starch. Hemicellulose represents an average concentration of 5.87%, followed by lignin (2.03%–2.64%), cellulose (1.33%–2.75%), resistant starch (1.58%–1.90%), pectin (0.45%–1.20%), 1-Kestose (0.21%–0.36%), Nystose (0.11%–0.23%), and 1F-Fructofuranosylnystose (0.12%–0.22%).

Additionally, fructans, galactan, and homogeneous heteropolysaccharides are the main active polysaccharide in PCH (Gao et al., 2024; Liang et al., 2021; Zhang et al., 2021). The pharmacological properties of these polysaccharides depend on their structural characteristics (Yang et al., 2019).

## 4 Factors affecting the composition of polysaccharides

Raw rhizomes of PCH are rarely used without processing. The traditional Chinese pharmaceutic technique known as *Paozhi* is employed to prepare these rhizomes for clinical use, is accordance with Traditional Chinese Medicine (TCM) theory (Sheridan et al., 2015). *Paozhi* results in alterations of nutrient content and medicinal properties, enhancing efficacy, reducing toxicity, and improving herbal flavor (Wu et al., 2018). These transformations make herbal medicines more effective and suitable for personalized therapy (Wu et al., 2018).

Extensive texts recorded *Paozhi* methods for *P. cyrtonema* Hua rhizomes, typically containing cutting, wine-steaming, wine-stewing, steaming, drying, nine times steaming and nine-basking, and copying (Deng et al., 2020; Liu J. et al., 2022; Qin, 2018; Zhang X. N. et al., 2024). Repeated steaming and drying can enhance the tonic function of raw rhizomes (Jin et al., 2018). Currently, these methods are widely used to optimize the medicinal properties of the rhizomes.

### 4.1 Steam and drying processing

Steaming is widely employed to process rhizomes that contain large accounts of carbohydrates, mucilage, and starch, including *Gastrodiae Rhizoma*, *Curcumae Rhizoma*, *Asparagi Radix*, *Glehniae Radix*, and *Polygonati Rhizoma*. It serves as a distinct preparation method for numerous TCM herbs prior to drying. Drying is one of the oldest and most efficient techniques for preserving herbal parts, significantly impacting the quality of herbal products, particularly in terms of flavor and color. Classical sun-drying is gradually being supplanted by advanced methods, including microwave drying, infrared drying, and heated-air drying (Zhu et al., 2022). These modern methods offer controlled temperatures, shorter drying times, and are not influenced by climatic conditions, thus maintaining the quality of the dried products (Zhu et al., 2022).

#### 4.1.1 Affecting decomposition of polysaccharides

**FIGURE 2:**
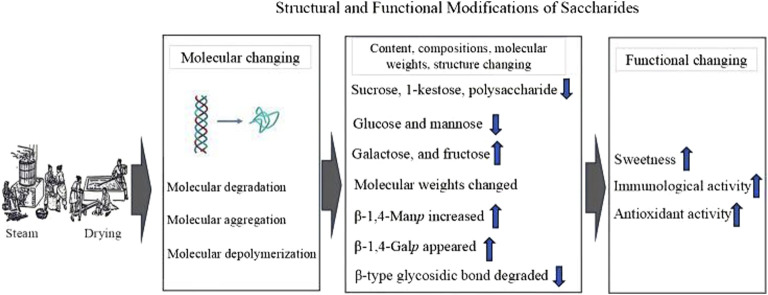
The steam and drying processing impact the *Polygonatum cyrtonema* Hua polysaccharide content, compositions, molecular weights, structure, and activities.

Steam and drying processing significantly impact the polysaccharide content, composition, molecular weights and structure due to molecular degradation, aggregation, and depolymerization (Wu et al., 2022) (Figure 2). These changes, in turn, affect their activities, such as immunological and antioxidant functions. Steaming significantly decreases the polysaccharide content in PCH (Wu et al., 2022). This reduction is likely due to the decomposition of polysaccharides into monosaccharides during the steaming process (Jin et al., 2018). It results in an increase in fructose, galactose, and glucose contents, and a reduction in polysaccharides, 1-kestose, and sucrose contents (Jin et al., 2018). The elevated fructose level is identified as the primary factor responsible for the increased sweetness after steaming (Jin et al., 2018).

#### 4.1.2 Affecting molecular weights

Molecular weights of polysaccharides could change during steaming. Initially, molecular weights increase due to depolymerization, but with further steaming, they decrease (Chen Z. et al., 2022). A study demonstrated that polysaccharides (MW is 4.35 × 10^3^ Da) from crude PCH were primarily composed of fructose, with minor quantities of glucose (Teng et al., 2023). In contrast, polysaccharides (MW is 4.24 × 10^4^ Da) from steam-processed PCH consisted of glucuronic acid, galacturonic acid, mannose, xylose, glucose, arabinose, and fructose (Teng et al., 2023).

#### 4.1.3 Affecting glycosidic bond

In terms of structure, polysaccharides from raw PCH contain β-type glycosidic bonds, while steaming first degrades the β-type fructofuranose in PCHP (Wu et al., 2022). This process elevates galactose levels while reducing glucose and mannose levels, resulting in the appearance of β-1,4-Gal*p* and an increase in β-1,4-Man*p*, which enhances antioxidant activity (Chen Z. et al., 2022). Polysaccharides from crude PCH exhibit a triple-helical conformation, whereas those from steam-processed PCH form a random coil (Teng et al., 2023). Moreover, steamed PCHP has a stronger effect in preventing oxidative damage (Teng et al., 2023). PCH steamed for 2–4 h demonstrates higher immunological activities than raw rhizomes (Wu et al., 2022). However, longer steaming times (6–12 h) cause excessive degradation of PCH, negatively impacting their immunological activities (Wu et al., 2022).

### 4.2 Nine-steam-nine-bask

Steam processing has gradually evolved into the “nine-steam-nine-bask” method, involving 9 cycles of steaming and sun-drying (Qin, 2020; Zheng et al., 2022; Zhu et al., 2022). For detoxification, enhancing efficacy, altering meridians, facilitating storage, and eliminating bacteria, PCH rhizomes are typically processed using this “nine-steam-nine-bask” method (Qin, 2020; Wang M. et al., 2024), which is now more widely used than other methods (Yao et al., 2022). Research has exhibited that this method could increase the total phenol content while lowering polysaccharide concentration, as well as enhancing the antioxidant activity of PCH (Wang X. et al., 2024). Some researchers consider that the term “nine” in this context is often interpreted to mean “multiple times” rather than strictly nine cycles (Liu J. et al., 2022). The number of cycles varies based on the type of herbs used. The primary goal is to enhance the medicinal properties by increasing beneficial ingredients and reducing harmful ones. Consequently, many researchers have investigated the optimal number of processing cycles to achieve the desired outcomes.

**FIGURE 3:**
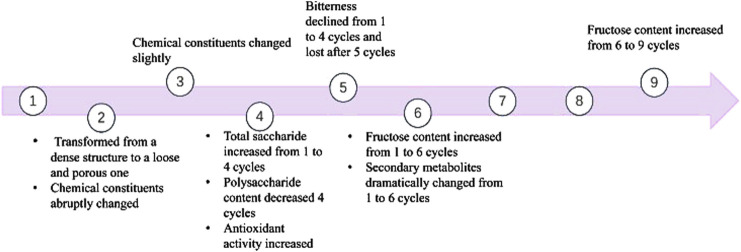
The changes of saccharides and *Polygonatum cyrtonema* Hua during “nine-steam-nine-bask” processing.

During the classical *Paozhi* method (nine times steaming and nine times sun-drying), significant alterations in taste, constituents, and drug efficacy of PCH occur depending on the number of processing cycles (Figure 3). Notably, after five cycles of alternating steaming and solarization, the rhizomes of PCH exhibited an almost complete loss of bitterness, comparable to those processed through nine cycles of steaming (Nie et al., 2024). Furthermore, after just two cycles, the rhizomes transformed from a dense structure to a loose and porous one (Yao et al., 2022).

The chemical composition in the crude PCH and processed PCH were nearly identical after one cycle of steaming and basking (Nie et al., 2024). However, abrupt changes occurred during the second cycle, with further noticeable differentiation after the third cycle (Nie et al., 2024). Results demonstrated that the total saccharide content increased after 1–4 cycles of “nine-steam-nine-bask” but decreased from four to nine cycles (Zhu et al., 2022). Similarly, fructose content increased from one to six cycles, then decreased from six to nine cycles (Zhu et al., 2022). Polysaccharide content gradually decreased with repeated steaming and basking, stabilizing after the fourth cycle (Fan et al., 2020; Mei et al., 2025).

In summary, considering polysaccharide content and chemical constituents as key evaluation indices, 4 cycles of “nine-steam-nine-bask” emerge as a favorable postharvest processing method for achieving optimal flavor, taste, and efficacy.

### 4.3 Wine and honey processing

Sixteen differential components, including kingianoside Z (a saponin), disporopsin (a steroidal saponin), and linoleic acid (a polyunsaturated fatty acid), were identified, revealing significant differences between raw and wine-processed PCH dried rhizomes (Ren et al., 2021). Processing with huangjiu and honey darkened the rhizomes from bright yellow, and polysaccharide content decreased from 7.42 g/100 g–3.12 g/100 g (huangjiu-steamed) and 4.41 g/100 g (honey-steamed) (Deng et al., 2024).

### 4.4 Black beans processing

Processing *P. cyrtonema* Hua with black beans, as recorded in the Processing Standard of Chinese Herbal Pieces in Sichuan Province (2015 Edition), induces significant changes in its chemical composition (Sichuan Food and Drug Administration, 2015). The amount and concentration of low-polarity compositions elevate notably, whereas medium-polarity compositions either reduced or vanished entirely (Zhang J. et al., 2023). The average polysaccharide content rises from 12.19% to 32.28% in processed samples (Zhang J. et al., 2023).

### 4.5 Fermentation

Fermentation has minimal impact on the polysaccharide content of PCH. In raw PCH, neutral polysaccharides dominate, comprising 68.48%. After fermentation with *Bacillus subtilis*, neutral polysaccharides account for 64.10%. When fermented with *Saccharomyces cerevisiae*, the disparity between acidic and neutral polysaccharides narrows, with 52.20% and 47.80%, respectively (Cheng et al., 2023).

### 4.6 Other factors influencing components

PCH is planted in forests, avoiding the use of arable land (Si and Zhu, 2021). Besides processing, the nutrient content and phytochemicals in PCH vary by region (Gong Q. et al., 2024; Song et al., 2023; Zhang D. T. et al., 2023), indigenous rhizosphere microorganisms (Haiyan et al., 2024; Yang L. et al., 2024; Zhou et al., 2023), pathogenic fungi (Wei et al., 2024; Yin et al., 2024), different germplasms (Jiang et al., 2023; Zheng et al., 2023), age (Li D. et al., 2022; Wang N. et al., 2023), agricultural practices (Jun’an et al., 2022; Tang et al., 2022), and extraction methods (Liu J. et al., 2023). Identifying the geographical origin of PCH is crucial due to significant quality and market value variations arising from different growing environments and climatic conditions, posing challenges for its industrial and medicinal applications (Gong Q. et al., 2024; Hao et al., 2024). The accumulation of bioactive compounds and chemicals in PCH varies with growth stages, with polysaccharide content increasing with age but decreasing after a certain period (Wu et al., 2024). Moreover, material-fluid ratio and extraction temperature significantly affect the microstructure of polysaccharide molecules, as well as their viscosity, radius of gyration, diffusion coefficient, mean square displacement, radial distribution function, and hydrogen bond properties, impacting their dissolution and diffusion (Liu J. et al., 2023).

## 5 Health benefits and the underlying mechanism

### 5.1 Health benefits of polysaccharides

Polysaccharides, a key active ingredient in TCM, possess multifaceted health benefits, such as anti-inflammatory, immunomodulatory antioxidant, anti-aging, modulating the gut microbiota, preventing cardiovascular disease, anticancer, anti-fatigue, anti-obesity, glycemic regulation, and anti-depression effects. The versatile activities including anti-inflammatory, anti-obesity, anti-fatigue, immunomodulatory, antioxidant, modulation of the gut microbiota and preventing cardiovascular disease.

#### 5.1.1 Anti-inflammatory effects

The anti-inflammatory activity studies of *P. cyrtonema* Hua polysaccharides (PCHPs) have demonstrated significant therapeutic potential. PCHPs effectively inhibited the growth of MH7A cells through reducing IL-6 and IL-1 contents while elevating IL-10 (Wang Z. et al., 2023). This was achieved through the induction of apoptosis in synovial fibroblasts, evidenced by a decrease in MH7A cells in the G2 phase and an increase in the G0/G1 phase (Wang Z. et al., 2023). Consequently, PCHPs were speculated to exert their anti-inflammatory effects by arresting the cell cycle in the G0/G1 phase (Wang Z. et al., 2023). Additionally, polysaccharides inhibited LPS-induced M1 polarization of macrophages and promoted their polarization to M2, further contributing to their anti-inflammatory properties (Zhou et al., 2022).

#### 5.1.2 Immunomodulatory effects

Immunomodulatory studies in RAW 264.7 cells revealed that PCHPs significantly stimulated cell proliferation, activated macrophages, and enhanced their phagocytic activity (Wu et al., 2022). PCHPs also elevated IL-6, TNF-α, and nitric oxide (NO) secretion, indicating a strong potential for immune enhancement (Wu et al., 2022). Moreover, neutral PCHP was found to enhance macrophage proliferation and phagocytosis, inhibit LPS-induced M1 polarization, and suppress M2 polarization induced by IL-13 and IL-4 (Wen et al., 2024). Neutral PCHP also suppressed IL-6 and TNF-α generation in both M1 and M2 cells while promoting the secretion of IL-10 (Wen et al., 2024). Additionally, PCHPs could markedly improve lung injury by reducing inflammatory factors in bronchoalveolar lavage fluid and lowering myeloperoxidase levels in lung tissue through the nuclear factor kappa-B (NF-κB) pathway (Gan et al., 2022). PCHPs also increased SOD levels, indicating enhanced antioxidant capacity via the 5′ adenosine monophosphate-activated protein kinase (AMPK)-Nrf2 pathway (Gan et al., 2022). These findings suggest that PCHPs could serve as potent immunomodulatory agents.

#### 5.1.3 Antioxidant and anti-aging effects

Research has indicated that PCHPs exhibit potent antioxidant and anti-aging properties. *In vitro* studies have shown that PCHPs effectively scavenge radicals, suggesting strong antioxidant activity (Zhao et al., 2023). Specifically, PCHPs demonstrated strong scavenging capacity for 2,2′-azino-bis (3-ethylbenzthiazoline-6-sulfonic acid) (ABTS), hydroxyl, and 1,1-diphenyl-2-picrylhydrazyl (DPPH) radicals with concentrations ranging from 0.2 to 1.0 mg/mL (Zuo et al., 2024). Additionally, studies have found that various types of PCHPs exhibit increasing anti-oxidative activity, achieving prominent scavenging effects against ABTS and DPPH radicals, with scavenging rates of 90.1% and 88.8%, respectively (Hu et al., 2022). Further, PCHPs attenuated reactive oxygen species (ROS) generation through up-regulating the Nrf2/heme oxygenase (HO) −1 pathway, thereby reducing neuronal-regulated cell death in H_2_O_2_-induced microglial injury and ferroptosis in microglia (Li J. et al., 2022).

In addition, PCHPs exhibited significant free radical-scavenging capacity *in vivo*. Administration of PCHP observably restored histopathological changes, reduced ROS production, and restored antioxidative enzymes activities in oxidative stress mice (Teng et al., 2023). In this study, PCHPs were found to enhance the Nrf2/heme oxygenase-1 (HO-1) antioxidant pathways, contributing to their antioxidative and anti-ferroptosis effects (Teng et al., 2023). Furthermore, PCHPs prolonged nematodes’ lifespan by enhancing their resistance to oxidative stress, UV irradiation, and heat stress (Wang W. et al., 2023; Zhang X. et al., 2024). They reduced intestinal lipofuscin accumulation, malondialdehyde (MDA) and ROS contents, while increasing antioxidant enzymes activities including SOD and catalase (CAT) (Wang W. et al., 2023; Zhang X. et al., 2024). The anti-aging of this polysaccharide was related to the downregulation of daf-2 and age-1 genes expression, while up-regulating aging- and oxidative stress-associated genes expression including hsp-16.2, sod-3, skn-1, and daf-16 genes expression in the insulin/insulin-like growth factor signaling pathway (Wang W. et al., 2023; Zhang X. et al., 2024). It further facilitated DAF-16 nuclear translocation (Wang W. et al., 2023; Zhang X. et al., 2024). Thus, polysaccharides from PCH have been shown their anti-aging effects, establishing a basis for future research on anti-aging.

In addition, PCHPs exhibited significant antioxidant activity contributing to their inhibitory effects against various bacteria, including *E. coli*, *Salmonella typhimurium*, *Bacillus subtilis*, and *Staphylococcus aureus* (Zheng et al., 2023). Moreover, the antibacterial effects of these PCHPs were more pronounced on Gram-positive bacteria (*S. aureus* and *B. subtilis*) compared to Gram-negative bacteria (*S. typhimurium* and *E. coli*) (Li et al., 2018a). These findings underscore the potential of PCHPs as natural antimicrobial agents with broad-spectrum antibacterial properties, particularly against Gram-positive bacteria.

#### 5.1.4 Preventing cardiovascular disease

Numerous studies have shown that polysaccharides from *PCH* have positive effects on cardiovascular diseases caused by various factors. In animal models, a high-fat diet (HFD) leads to atherosclerosis, characterized by severe dyslipidemia, atherosclerotic lesions, oxidative damage, and inflammation, with these effects being more pronounced in males than in females (Guo et al., 2024). Administration of a purified PCHP repaired these adverse variations, with greater intervention effects observed in male LDLr−/− mice compared to the female ones (Guo et al., 2024). Collectively, the results indicate that PCHP has the potential for preventing atherosclerosis and lowering lipid levels.

In terms of the mechanism, the sweet taste receptor T1R2/T1R3 directly recognizes PCHP playing an essential role in activating the T1R2/T1R3-mediated cyclic adenosine monophosphate (cAMP) signaling pathway (Xie et al., 2020). Then, PCHP mediates the development of atherosclerosis by three pivotal signaling pathways (NF-κB, mitogen-activated protein kinase (MAPKs), and protein kinase B (Akt)) (Guo et al., 2024). This was evidenced by the inhibitory effects on activation of extracellular-regulated kinase 1/2 (ERK1/2), Akt, p38, p65, and NF-κB (IκB) in atherosclerotic mice (Guo et al., 2024). Moreover, PCHP effectively promotes glucagon-like peptide-1 (GLP-1) generation which is beneficial for protecting cardiovascular health (Xie et al., 2020). Based on current investigations, the protective mechanism of PCHP in preventing atherosclerosis and delaying the onset of cardiovascular disease by inhibiting NF-κB/MAPKs/Akt-mediated inflammatory responses and activating the cAMP signaling pathway.

#### 5.1.5 Anticancer

Four polysaccharides extracted from PCH, namely CASS, DASS, CHSS, and HBSS, were extracted using concentrated alkali, diluted alkali, chelating agent, and hot buffer, respectively (Li et al., 2018b). The primary monosaccharides of the four heteropolysaccharides were identified as galactose, mannose, arabinose, and rhamnose (Li et al., 2020). The effects of these PCHPs on apoptosis, cell cycle, caspase-3 activity, cytotoxicity, and proliferation inhibition of human cervical cancer HeLa cells were evaluated. The inhibition rates were ranked as DASS < CHSS < HBSS < CASS, with the maximum inhibiting rate reaching 74.45% (Li et al., 2020). Cell cytotoxicities were evaluated in the order of DASS < HBSS < CHSS < CASS, with the highest death rate being 82.47% (Li et al., 2020). Caspase-3 activities were activated by CHSS < DASS < HBSS < CASS, with the highest induction being 2.95-fold (Li et al., 2020). These four PCHP types also exhibited notable antioxidant and antibacterial activities (Li et al., 2020).

Heteropolysaccharides from PCH arrested the cell cycle at the G2/M phase through upregulating the expression of Wee1, p53, p21, and CyclinD1 genes, while downregulating cyclin-Dependent Kinase (CDK)-1, CyclinB1, Wee1, checkpoint kinase 2 (CHK2), and Survivin genes expression. Additionally, the heteropolysaccharides increased various genes expression in the death receptor pathway, including death receptor (DR)3, DR5, FasL, caspases-8, and caspase-10, TNF Receptor-Associated Death Domain (TRADD), TNF-R1, TNF-α, Fas-Associated protein with Death Domain (FADD), Poly (ADP-Ribose) Polymerase (PARP), as well as several proteins expression including caspases-8, -10 and FasL (Li et al., 2020). Conversely, it decreased anti-apoptotic genes (Bcl-xL and Bcl-2) and protein (Bcl-2) expressions. In the mitochondrial pathway, CASS upregulated the expression of pro-apoptotic genes (caspases-9, -7, and -3, Puma, Cytc, and Bak) and caspases-3 and -9 protein expression, leading to cell apoptosis (Li et al., 2020). Moreover, the effects on improving lipid disorders and antioxidant in palmitic acid-induced HepG2 cells revealed additional potential anti-cancer mechanisms (Jin et al., 2024; Zuo et al., 2024).

#### 5.1.6 Anti-fatigue effect

Polysaccharides from PCH exhibit anti-fatigue activity by preventing excessive accumulation of metabolites, reducing muscle damage, delaying oxidative injury, and modulating gut microflora (Wang N. et al., 2023). Studies have shown that PCHPs significantly extended the swimming time of exhausted mice, along with decreasing MDA, glutathione peroxidase (GSH-Px), SOD, blood urea nitrogen (BUN), and lactic acid (LA), while increasing muscle glycogen and liver glycogen levels, thereby promoting ATP production (Shen et al., 2021).

The mechanism for this anti-fatigue was related to enhancing osteocalcin-mediated interaction between muscles and bones. It significantly stimulated bone marrow stromal Cells (BMSC) differentiation into osteoblasts and enhanced muscle fibers’ cross-sectional area (Li X. L. et al., 2023; Shen et al., 2021). Consequently, PCHP increased the energy metabolism of myoblast through elevating osteocalcin generation in the skeleton (Li X. Y. et al., 2023; Shen et al., 2021). This caused an elevation in the protein expression of G-protein coupled receptor family C group 6 member A- *Homo sapiens* (GPRC6A), Runt-related transcription factor 2 (Runx2), phosphor-Smad1, and bone morphogenetic protein-2 (BMP-2); the phosphorylation levels of hormone-sensitive lipase (HSL) and cAMP response element-binding protein (CREB); and the mRNA levels of carnitine palmitoyltransferase 1B (CPT1B), fatty acid transport Protein 1 (FATP1), cluster of differentiation 36 (CD36), glucose transporter type 4 (GLUT4), and thus boosting ATP generation (Li X. L. et al., 2023; Shen et al., 2021). Thereby, PCHP alleviated fatigue in swimming-exhausted mice by regulating osteocalcin signaling (Li X. Y. et al., 2023).

In chemotherapy-induced cachectic mice, PCHP markedly mitigated the weight loss of organs and muscles, thereby alleviating muscle fiber atrophy (Tang et al., 2023). An increased pro-inflammatory factor interleukin-6 (IL-6) and a decreased serum immunoglobulin level were reversed by PCHP (Tang et al., 2023). PCHP also promoted the viability of C2C12 cells, increased the diameter and fusion index of the myotubes, and improved C2C12 myotube atrophy induced by cisplatin (Xueyang et al., 2023). The anti-atrophy mechanism of PCHP involved maintaining protein metabolism homeostasis in the gastrocnemius muscle through the IL-6/signal transducer and activator of transcription 3 (STAT3)/cathepsin L (CTSL) and Diacylglycerol kinase (DGKζ)/the forkhead box O-class (FoxO)/Atrogin1 signaling axis, mediating the ubiquitin-proteasome and autophagy-lysosome systems, and downregulating the accumulation of ceramide via sphingomyelin phosphodiesterase 2 (Tang et al., 2023).

#### 5.1.7 Anti-obesity

PCHP could markedly reverse insulin resistance as indicated by a reduction in obesity markers, enhanced leptin and fasting blood glucose levels, and improvements in serum lipid profiles. In obese mice induced by high-fat-diet, PCHP demonstrated a preventive effect against obesity through modulating the metabolism of glycerol phospholipids, arachidonic acid, and linolenic acid (Liang et al., 2022). It also reduced hepatic lipid droplet infiltration and adipocyte size in adipose tissues of these obese mice (Liang et al., 2021). The anti-obesity mechanism of PCHP involved significantly downregulating fatty acid synthase (FAS), sterol regulatory element-binding protein-1c (SREBP-1c), peroxisome proliferator-activated receptor-γ (PPARγ), and CCAAT/enhancer-binding protein-α (C/EBPα) gene expression, while increasing levels of carnitine palmitoyltransferase 1 (CPT1) and uncoupling protein 2 (UCP2), which are strongly associated with glucolipid metabolism and thermogenesis (Liang et al., 2021). In non-alcoholic fatty liver disease (NAFLD) mice, PCHP mitigated hepatic pathological damage, and improved abnormal lipid metabolism and oxidative stress, promoted SCFA generation, and balanced the intestinal microbiota composition (Liu W. et al., 2022). These results provide evidence that PCHP is a promising agent for obesity prevention and treatment.

#### 5.1.8 Glycemic regulation effect

In hyperglycemic conditions, high-dose treatment with PCHP promoted survival rates and reduced blood glucose levels. This polysaccharide significantly improved liver structural disorders, hepatocyte degeneration, and active hepatocyte infiltration in type 1 diabetic mice by decreasing IL-6 and IL-1β levels, while increasing insulin receptor substrate 1 gene expression in the diabetic liver (Lu et al., 2020). Supplementation with a combination of konjac glucomannan and PCHP has shown significant efficacy in improving long-term glucose metabolism through various metabolic pathways. This supplementation resulted in improved overall glucose regulation, insulin levels, and fasting blood glucose (Chang et al., 2024). Additionally, the combination enhanced body weight, liver health, and lipid homeostasis, more effectively than either component alone (Chang et al., 2024). These finding suggests that PCHP can be strategically utilized as a hypoglycemic component in nutritional management and glycemic regulation.

#### 5.1.9 Anti-depression

In TCM theory, post-traumatic stress disorder (PTSD) is considered a form of depression syndrome, often associated with kidney and heart deficiencies. *PC*H is known to replenish Qi and blood and tonify the five zang organs, leading to its widespread use in TCM prescriptions for treating depressive syndromes. In cellular (HT-22 cell) experiments, *PCHP* attenuated reactive oxygen species (ROS) generation induced by lipopolysaccharide (LPS) (Shen et al., 2022). In an animal model of depression, LPS induced a significant alteration in animals indicated by an elevated levels in glial fibrillary acidic protein (GFAP), NF-κB, p-ERK, Iba1, cleaved-caspase-1, caspase-1, nod-like receptor protein containing pyrin 3 (NLRP3), apoptosis-associated speck-like protein containing a caspase recruitment domain (ASC), and calpain-1, and a reduced production in Nrf2, suprachiasmatic nucleus circadian oscillatory protein (SCOP), phosphatase and tensin homolog (PTEN), and calpastatin (Shen et al., 2022). Administration of P*CHP* reversed these changes, and decreased proinflammatory cytokine secretion (Shen et al., 2022). Moreover, in another model, mice induced by single prolonged stress, PCHP could prevent PTSD-like characteristics, such as heightened anxiety-related behaviors and the acquisition of fear memories (Xie et al., 2024).

In addition, PCHP treatment counteracted the decreased levels of GluA1, HO-1, activity-regulated cytoskeleton-associated protein, phospho-tyrosine kinase receptor B, Nrf2, postsynaptic density protein 95, and brain-derived neurotrophic factor (Xie et al., 2024). It also mitigated the increased expression of apoptosis-associated speck-like protein, Glutamate Ionotropic Receptor NMDA Type Subunit 2B (GluN2B), and NLRP3 (Xie et al., 2024). In addition, PCHP prevented depression-like behavior by Nrf2 and NLRP3 signaling pathways(Shen et al., 2022). These findings provide evidence that PCHP has anti-depressant properties through the oxidative stress-calpain-1-NLRP3 signaling pathways, potentially through a mechanism dependent on the Nrf2/HO-1 signaling pathway.

### 5.2 Mechanisms underlying the biological activities

#### 5.2.1 Antioxidant mechanism

**FIGURE 4:**
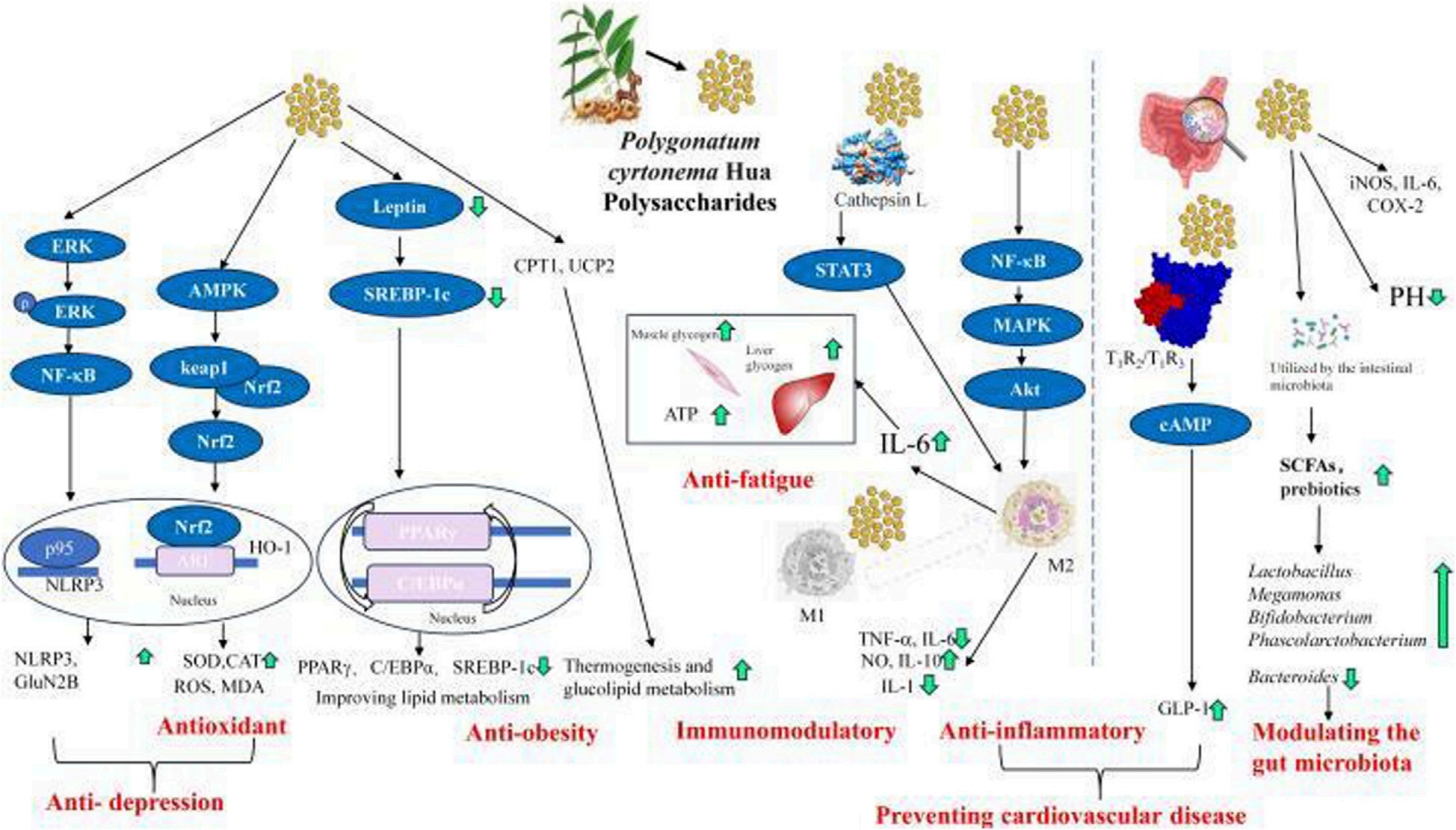
The versatile activities including anti-inflammatory, anti-obesity, anti-fatigue, immunomodulatory, antioxidant, Modulating the gut microbiota and preventing cardiovascular disease, and the underlying mechanisms of *Polygonatum cyrtonema* Hua polysaccharides. ERK, extracellular-regulated kinase; NF-κB, nuclear factor kappa-B; AMPK, 5′ adenosine monophosphate-activated protein kinase; Nrf2, nuclear factor erythroid 2-related factor 2; ARE, Antioxidant Response Element; NLRP3, nod-like receptor protein containing pyrin 3; HO-1, heme oxygenase-1; SOD, superoxide dismutase; CAT, catalase; ROS, reactive oxygen species; MDA, malondialdehyde; SREBP-1c, sterol regulatory element-binding protein-1c; PPARγ, peroxisome proliferator-activated receptor-γ; C/EBPα, CCAAT/enhancer-binding protein-α; CPT1, carnitine palmitoyltransferase 1; UCP2, uncoupling protein 2; STAT3, signal transducer and activator of transcription 3; IL, interleukin; TNF-α, tumor necrosis factor-α; NO, nitric oxide; MAPK, mitogen-activated protein kinase; Akt, protein kinase B; cAMP, cyclic adenosine monophosphate; GLP-1, glucagon-like peptide-1; iNOS, inducible nitric oxide synthase; COX-2, cyclooxygenase-2; SCFAs, short-chain fatty acids.

PCHP exhibit antioxidant activity primarily by modulating the Keap1-Nrf2-ARE signaling pathway. Nrf2 is bound to Keap1 in the cytoplasm of normal cell. Exposure to PCHP disrupts this interaction, primarily by activating AMPK upstream (Figure 4). This allows Nrf2 to translocate into the nucleus, where it binds AREs and enhances transcription of antioxidant enzymes such as SOD, GPX, HO-1, and CAT. These enzymes collectively scavenge ROS and reduce oxidative damage.

#### 5.2.2 Antidepressant pathways

The antidepressant effects of PCHP involve modulation of several key signaling pathways (Figure 4). PCHP enhances ERK phosphorylation, which activates NF-κB and promotes the interaction between p95 and NLRP3, resulting in upregulation of NLRP3 and GluN2B expression. These effects are closely linked to its antioxidant activity, particularly via the AMPK-Keap1-Nrf2-ARE pathway. Together, these findings suggest that PCHP’s antidepressant action results from direct modulation of neuronal and inflammatory signaling as well as indirect neuroprotection through enhanced antioxidant defenses.

#### 5.2.3 Anti-obesity mechanism

The anti-obesity effects of PCHP are associated with the regulation of lipid metabolism and energy expenditure via multiple molecular pathways (Figure 4). A key mechanism is the downregulation of leptin, which reduces SREBP-1c expression, subsequently inhibiting downstream targets such as PPARγ and C/EBPα. By suppressing these factors, PCHP inhibits adipogenesis and promotes lipid catabolism. Additionally, PCHP upregulates CPT1 and UCP2, increasing energy expenditure. These combined actions suggest a dual mechanism of anti-obesity activity: inhibition of lipogenesis and adipocyte differentiation, alongside enhanced lipid oxidation and thermogenesis, contributing to improved glucolipid metabolism and energy balance.

#### 5.2.4 M2-mediated effects

M2 macrophages play a key role in mediating the effects of PCHP, including anti-fatigue, immunomodulation, anti-inflammation, and cardiovascular protection (Figure 4). PCHP binds to Cathepsin L, regulating STAT3 signaling to activate M2 macrophages. It can also influence M1 macrophages to promote M2 activation. Additionally, M2 polarization is induced via the NF-κB-MAPK-Akt pathway. Activation of M2 macrophages increases IL-6 production, which enhances muscle and liver glycogen stores as well as ATP levels, contributing to anti-fatigue effects. M2 activation also balances the release of cytokines such as TNF-α, IL-6, NO, IL-10, and IL-1, thereby exerting anti-inflammatory and immunomodulatory effects. These anti-inflammatory actions are closely linked to the prevention of cardiovascular disease. Moreover, PCHP binds to T1R2/T1R3 receptors, activating cAMP signaling and promoting the release of GLP-1, further supporting cardiovascular health.

#### 5.2.5 Anticancer pathways

**FIGURE 5:**
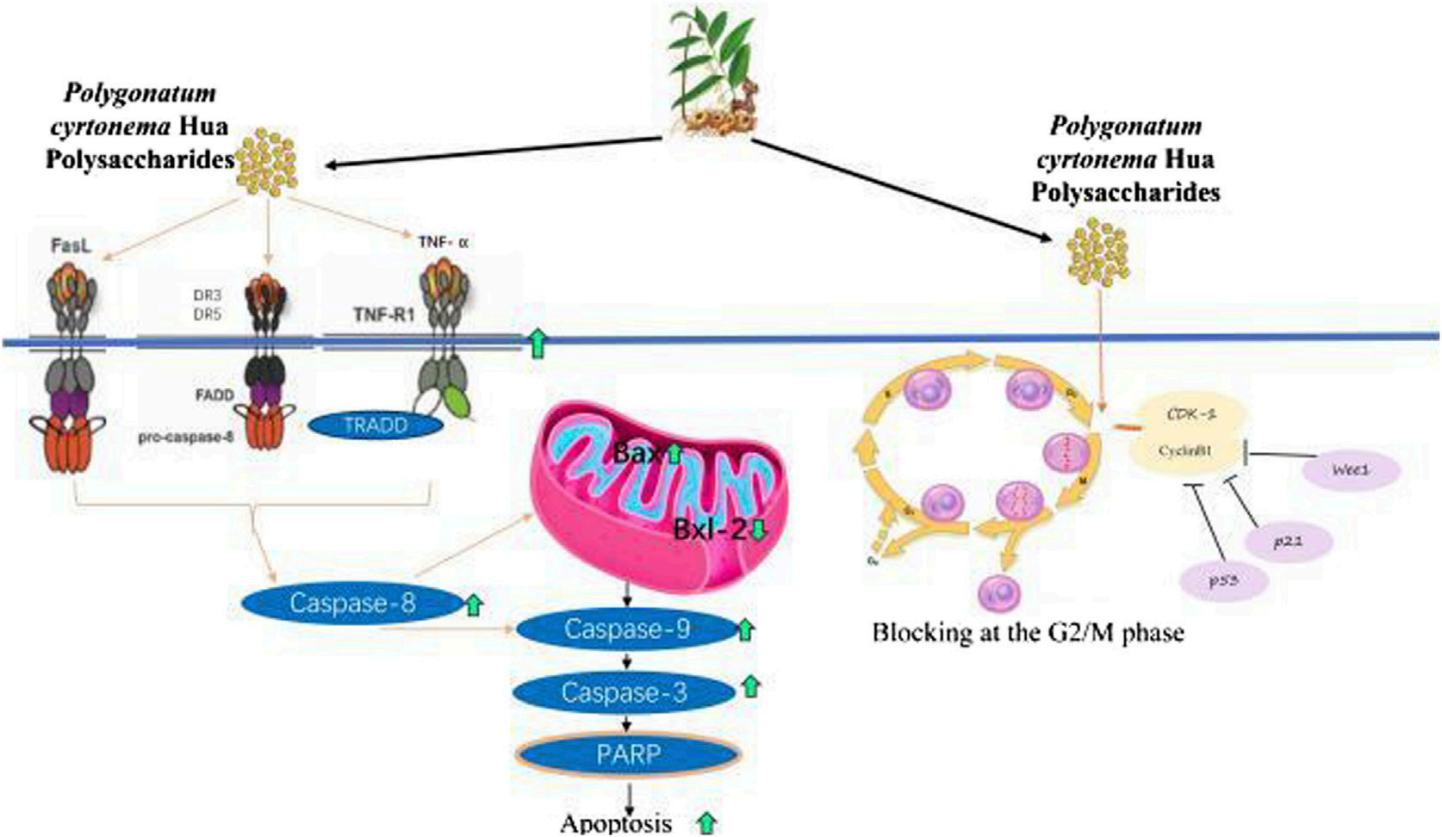
The anti-tumor mechanisms of *Polygonatum cyrtonema* Hua polysaccharides and lectin. DR, death receptor; FADD, Fas-Associated protein with Death Domain; TNF-α, tumor necrosis factor-α; TNF-R, TNF Receptor; TRADD, TNF Receptor-Associated Death Domain; PARP, Poly (ADP-Ribose) Polymerase.

PCHP’s anticancer pathways involve the induction of apoptosis via activation of the death receptor pathway (Figure 5). This is achieved through upregulation of death receptors DR3, DR5, FasL, and TNF-R1 on the cell membrane, leading to increased caspase-8 expression. Activated caspase-8 subsequently triggers pro-apoptotic caspases-9, -7, and -3, while downregulating anti-apoptotic proteins Bcl-xL and Bcl-2, thereby promoting apoptosis. Moreover, PCHP induces cell cycle arrest at the G2/M phase by upregulating Wee1, p53, p21, and Cyclin D1, while suppressing CDK1, Cyclin B1, CHK2, and Survivin expression.

#### 5.2.6 Modulating the gut microbiota

Previous research has demonstrated the significant potential of PCHPs in modulating gut microbiota. Intestinal health critically influences human’s mental state, immunity and metabolism (Rea et al., 2020). The human gastrointestinal system lacks enzymes capable of degrading certain carbohydrates (Wardman et al., 2022). Consequently, low molecular weight polysaccharides easily arrive in the colon, where they undergo fermentation and are used by the gut microorganism (Lin and Huang, 2022). This process helps maintain microecological balance and intestinal microbiota diversity. Additionally, non-starch polysaccharides serve as substrates for gut bacteria, bypassing digestion in the upper digestive tract (Reid et al., 2024). Thus, PCHPs could resist digestion, reaching the gut intact (Gao et al., 2024). During intestinal digestion, these polysaccharides undergo fermentation and are used by colonic microflora, producing prebiotics and short-chain fatty acids (SCFAs) (Álvarez-Mercado and Plaza-Diaz, 2022). This activity influences the colonic micropopulation and promotes host health (Álvarez-Mercado and Plaza-Diaz, 2022). *In vitro* fermentation of PCHPs notably decreased pH levels and increased SCFAs production, confirming their utilization by intestinal flora (Cheng et al., 2024; Gao et al., 2024). In another study, PCHP treatment significantly optimized body weight, repaired intestinal epithelial mucosal injury (Lin et al., 2024). It also alleviated oxidative stress and balanced inflammation response (Lin et al., 2024).

Mechanically, the anti-inflammatory, intestinal antioxidant defense, and immunomodulatory effects of polysaccharides may contribute to their ability to modulate gut microbiota homeostasis (Yuan et al., 2025). PCHPs have been shown to suppress inflammatory cytokines overproduction, including IL-6, cyclooxygenase-2 (COX-2), and inducible nitric oxide synthase (iNOS), in colitis mice induced by dextran sodium sulfate (Gong H. et al., 2024). PCHPs treatment significantly restored the intestinal barrier (occludin and zonula occludens-1) and mitigated colonic injury, regulating the gut microflora (Gong H. et al., 2024). This regulation decreased harmful bacteria, including *Klebsiella*, *Escherichia-Shigella* and *Bacteroides*, increased the beneficial bacteria, including *Lactobacillus*, *Megamonas*, *Bifidobacterium* and *Phascolarctobacterium*, and thereby alleviating colitis symptoms (Cheng et al., 2024; Gao et al., 2024; Gong H. et al., 2024) (Figure 4). Overall, PCHP can enhance body’s health by balancing the intestinal microbiota, making it a promising candidate for development as a functional food.

## 6 Potential application

Polysaccharides have seen widespread use in the pharmaceutical and nutraceutical areas (Zhang J. G. et al., 2024).

### 6.1 Medicine

#### 6.1.1 Chronic metabolic disease

Based on the aforementioned health benefits, PCH and its polysaccharides demonstrate antioxidative, anti-inflammatory, immunomodulatory, and anti-ferroptosis effects. These properties present new avenues for protecting human health against accumulated ROS in the central nervous system (CNS) and cardiovascular system. The polysaccharide from PCH can be rapidly absorbed into the bloodstream after oral and intraperitoneal administration and is primarily distributed in the heart, spleen, and lungs, indicating targeted effects on these tissues (Gao et al., 2024). Consequently, PCHP is a promising candidate for treating CNS and cardiovascular diseases. Additionally, PCH can serve as a natural agent for treating NAFLD, alleviating fatigue, preventing diet-induced obesity, and combating aging. Furthermore, polysaccharides from PCH show promise as potential agents for cancer therapy.

#### 6.1.2 Intestinal disorders

Recent literature suggests that polysaccharides from TCM are promising agents for regulating the intestinal microbiota, thereby improving the body’s function, as well as enhancing disease resistance (Gao et al., 2024). Evidence indicates that several natural food-derived products, including dietary fiber, and polysaccharides, can mitigate pathological changes in ulcerative colitis by improving gut microflora and reducing intestinal inflammation (Lin et al., 2024; Xiao et al., 2024). These findings and the aforementioned pharmacological activities of PCHPs offer significant insights into the prevention and treatment of intestinal disorders.

### 6.2 Food

Studies have demonstrated various potential bioactive functions of PCH, highlighting its potential as a functional food, particularly as a prebiotic. PCH rhizoma contain abundant high-quality dietary fiber such as oligofructose and non-starch polysaccharides (Zheng et al., 2024). Dietary polysaccharides from PCH may significantly dampen glycemic responses to nutrients (Chang et al., 2024). Research indicates that PCHPs can balance gut microbiota, exhibiting potential prebiotic properties (Cheng et al., 2024). Moreover, galactan and fructan from PCH also exhibited notable prebiotic activity, enhancing *lactobacilli* and *bifidobacteria* growth (Zhang et al., 2021). These findings suggest that *P. cyrtonema* Hua and its polysaccharides could be considered as beneficial ingredients in food products, expanding their application as prebiotic with significant health benefits.

## 7 Future perspectives

### 7.1 Importance of clinical trials for validation

Despite the promising findings on the health benefits and therapeutic potentials of polysaccharides from PCH, several critical research gaps need to be addressed to fully harness their potential in clinical and food applications. *PCH* has traditionally been used in clinic based on TCM theory. To explore more beneficial effects of its active compounds, numerous studies have demonstrated antioxidant, anti-inflammatory, immunomodulatory properties, among others. However, there is a lack of comprehensive clinical trials to confirm these benefits in human subjects, as most current evidence comes from laboratory studies and animal models. To bridge this gap, well-designed clinical trials are essential to assess the effectiveness of PCH and its polysaccharides in humans.

### 7.2 Need for bioavailability and pharmacokinetic evaluation

Additionally, the bioavailability and pharmacokinetics of these polysaccharides compounds need to be thoroughly studied. Despite their beneficial effects, the processes of absorption, distribution, metabolism, and excretion of these compounds in the human body are not well understood. Research in this area is essential for developing high-efficiency dosage forms and ensuring consistent therapeutic outcomes.

### 7.3 Modification strategies for PCH polysaccharides

Researchers have found that liposomes can enhance the immunological activity of PCHP. Liposome-encapsulated PCHP promoted phagocytosis and proliferation of RAW264.7 cells, increased NO production, and stimulated the generation of related cytokines. Additionally, it elevated the levels and percentages of lymphocytes, platelets, and red blood cells in the bloodstream (Liu et al., 2024). It also mitigated thymus and spleen atrophy in immunocompromised mouse models (Liu et al., 2024). This research highlights the potential of modified PCHP for further studies, which could create more effective therapeutic agents.

### 7.4 Effects of processing methods

The pharmacological activities of polysaccharides can be influenced by various factors, particularly the methods of preparation and processing. This indicates that different preparation methods can yield varying levels of bioactivity. Many studies have attempted to demonstrate that reducing the cycles of the ‘Nine-Steam-Nine-Bask’ process could achieve similar polysaccharide contents while saving time. However, some researchers argue that the traditional “Nine-Steam-Nine-Bask” technique produces a product with unique and superior qualities that cannot be replicated by merely identifying phenotypic traits (Nie et al., 2024). Some studies have evaluated whether modern processing methods, including infrared and heated-air drying, could serve as potential substitutes for traditional methods. However, others have proved that these contemporary techniques are unsuitable for replacing the traditional steam-sun drying process (Zhu et al., 2022). Further research is needed to explore how these different processing methods impact the chemical composition and pharmacological properties of polysaccharides from PCH, ensuring that any modern adaptations do not compromise its therapeutic potential.

### 7.5 Structure–function relationships

In terms of mechanism, the molecular processes responsible for the therapeutic effects of polysaccharides are still largely unexplored. Scientists have been investigating how structure affects function in PCHP. Many researchers have studied the structure of PCHP, including their molecular weight, monosaccharide composition, main chain, and branches (Table 1). As showed in Table 1, the most active polysaccharides from PCH have low molecular weights ranging from 3.2 to 8.5 kDa, and the primary monosaccharide components include fructose, arabinose, ribose, galactose, galacturonic acid, rhamnose, mannose, and glucose.

**TABLE 1:** The molecular weight, monosaccharide species, main chain, and branches of polysaccharides from the rhizomes of *Polygonatum cyrtonema* Hua.

Type of polysaccharides	Molecular weight	Monosaccharide composition	Molar ratio of monosaccharides	Main chain and branches	References
Galactan	14.4 kDa			A backbone consisting predominately of 1,4-β-linked Gal*p* branched at the C-6 position by T-β-linked Gal*p*	Pan et al. (2025)
	8.5 kDa			→6)-β-D-Fru*f*-(2→, →1,6)-β-D-Fru*f*-(2→, →1)-β-D-Fru*f*-(2→, β-D-Fru*f*-(2→, and →6)-α-D-Gal*p*-(1→	Deng et al. (2024)
Fructans (agavin type fructan)				Two fructose chains, namely β-(2→6) and β-(2→1) fructosyl-fructose, attached to the sucrose core, and has branches of β-D-Fru*f* residues	Gao et al. (2024)
Homogenous polysaccharide	5.1 kDa	Galactose, mannose, rhamnose, galacturonic acid and glucose		α- and β-configurations	Liang et al. (2021)
				A (2→6) linked β-D-Fru*f* backbone with an internal α-D-Glc*p* and two (2→1) linked β-D-Fru*f* branches	Zhang et al. (2021)
Galactan				A (1→4)-β-D-galactan branched with a single β-D-galactose at C-6 at every nine residues	Zhang et al. (2021)
	3.2 kDa	Glucose and galactose	55.4%, 44.6%	*→*4)-α-D-Glcp-(1*→*4)-α-D-Glcp-(1*→* and *→*3)-β-D-Galp-(1*→*3)- β-D-Galp-(1*→* and *→*4)-α-D-Glcp-(1*→*1)-β-D-Galp-(3*→*	Wen et al. (2024)
Heteroglycan	37.46 kDa	Glucose, mannose, galactose, rhamnose, and galacturonic acid	3.5: 2.5: 1.3: 1.8: 0.8	→3)-*α-D-Glcp, →2)-α-D-Glcp (6→, →1)-β-D-Glcp (2→, →2)-α-D-GalAp (3,4→, →1) -β-D-Manp (3→, →2)-α-DGlcp (3→, side chains including →3)-α-D-Glcp, →2)-β-D-Galp (4→, →1)-β-D-Glcp (2→, →2,4)-α-D-Manp (6→, →3)-α-L-Rhap (4→ branches located at the O-3 position of →2)-α-D-GalAp* (3,4→	Zhao et al. (2023)
	8.77-1.84 ×10^3^ kDa	Mannose, galacturonic acid, glucose, galactose, xylose, and arabinose	13.8 : 3.5 : 22.7 : 2.7:1: 1.3		Wang et al. (2023c)
	3.350 kDa	Mannose, glucose, galactose, and arabinose	1:2.67:0.25:0.089		Zhang et al. (2023b)
	4.1 kDa			→1)-*β*-D-Fru*f*-(2→as the main chain, accompanied by side chains dominated by →6)-*β*-D-Fru*f*-(2→	Cheng et al. (2024)
		Fructose and glucose	28:1	→6)-*β*-*D*-Fru*f*-(2→, →1,6)-*β*-*D*-Fru*f*-(2→, →1)-*β*-*D*-Fru*f*-(2→, *β*-*D*-Fru*f*-(2→, and →6)-*α*-*D*-Gal*p*-(1→	Guo et al. (2024)
Homogenous polysaccharide				3-MeO- β-Glc*p*-(1→[→6)-α-Fru*f*-(2→]11→4)-α-Gal*p*	Wang et al. (2023c)
	4.65 kDa	Fructose, glucose, mannose, and galactose		→1)-β-D-Fruf-(2→ and →1,6)-β-D-Fruf-(2→, with small amounts of →6)-α-D-Glcp-(1→, →4)-β-D-Manp-(1→, and β-D-Glcp-(1→. The side chain is β-DFruf-(2→ linked at C-6 of →1,6)-β-D-Fruf-(2→	Liu et al. (2022a)
Fructans	5 kDa	Fructose and glucose		A (2→1)-linked *β*-D-fructofuranose (Fru*f*) backbone and (2→6)-linked *β*-D-Fru*f* side chains with an internal *α*-D-glucopyranose (Glc*p*) in neokestose form	Zhao et al. (2019)
		Rhamnose, arabinose, galactose, glucose, mannose, and fructose			He (2017)
		Glucose, mannose, rhamnose, galactose, ribose, and arabinose			Hu et al. (2021)
		Glucose, galactose, and mannose	1.73:0.58:1		Liu et al. (2023c)
Homogeneous	7.554 kDa	Mannose, galactose, glucose, galacturonic acid, arabinose, and rhamnose	33:13: 8:3.5:2:1		Xu et al. (2024)

**FIGURE 6:**
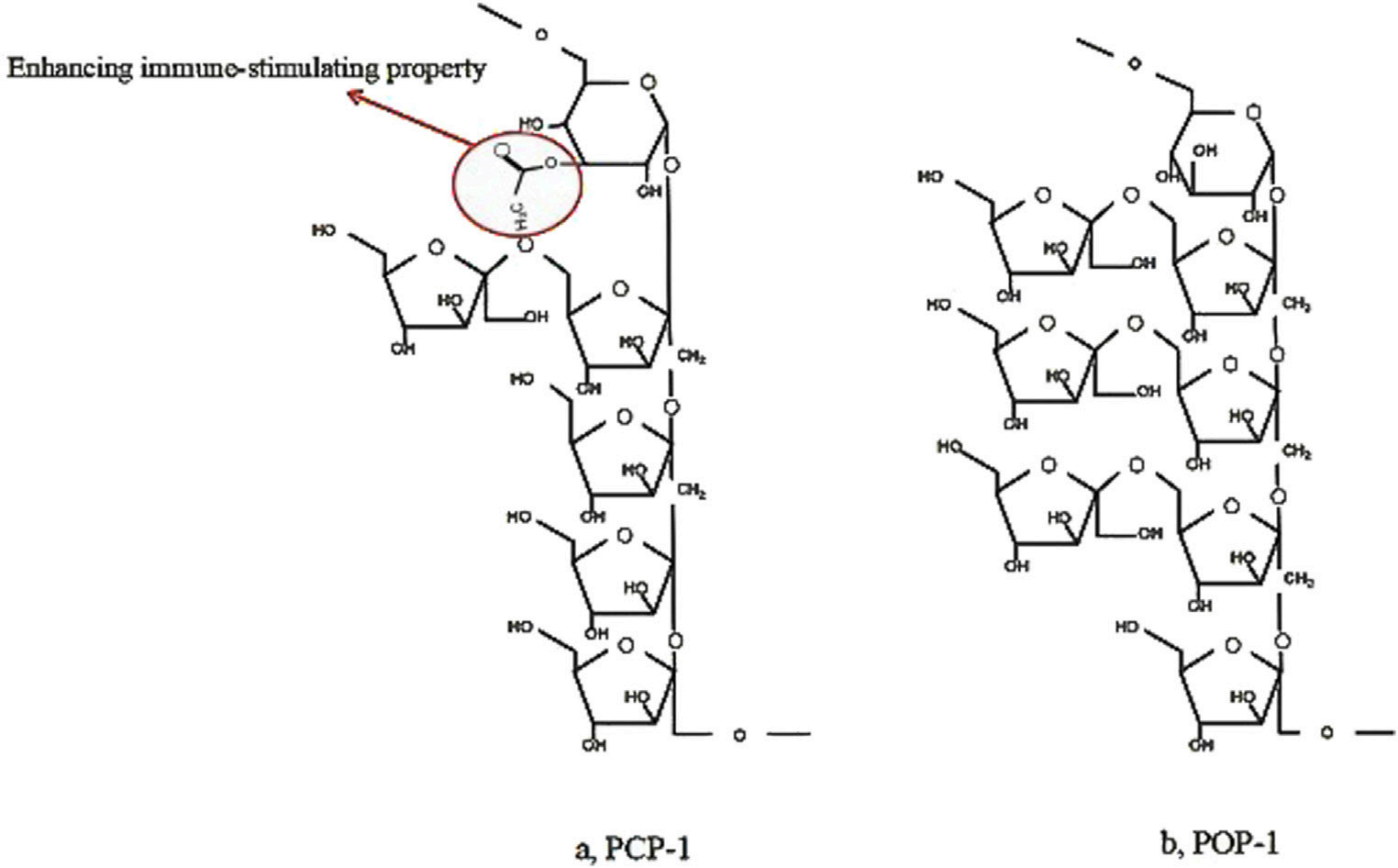
Comparison of two Representative polysaccharide structures from *Polygonatum cyrtonema* Hua (Zhao et al., 2019).

The bioactivity of these polysaccharides is closely related to their structural features (Table 1). For instance, complex fructan structures, such as β-(2→1) and β-(2→6)-linked fructofuranose (Fruf) chains attached to a sucrose core, provide multiple sites for microbial enzymatic action, promoting antioxidant and anti-inflammatory effects. Additionally, the presence of both α- and β-glycosidic configurations, as seen in neokestose-type linkages with internal α-D-glucopyranose (Glcp), may further enhance these bioactivities. Another illustrative example is provided by two PCH polysaccharides, named PCP-1 and POP-1, which were identified as consisting of glucose and fructose, with MWs of approximately 5 kDa (Zhao et al., 2019). Both PCP-1 and POP-1 had (2→1)-linked *β*-D-fructofuranose (Fruf) main chains and (2→6)-linked *β*-D-Fru*f* side chains, with an internal α-D-glucopyranose (Glc*p*) in neokestose form (Zhao et al., 2019). The distinction between PCP-1 and POP-1 lied in the presence of an acetyl group attached at the *O*-3 position of the α-D-Glc*p* residue in PCP-1, and PCP-1 had two fewer *β*-D-Fruf residues compared to POP-1 (Zhao et al., 2019) (Figure 6). Thus, PCP-1, which precedes POP-1, demonstrated immunoregulatory capacity in terms of IL-6 generation and cell survival rate in RAW264.7 cells (Zhao et al., 2019). The findings suggest that “acetyl group” in PCP-1 may enhance its immune-stimulating property. Understanding the structure-function relationship can pave the way for developing promising high-efficiency and targeted treatment.

### 7.6 Utilization of processing by-products

Finally, the current utilization of *P*. *cyrtonema* Hua primarily focuses on its cultivation for root harvesting, typically within 3–5 years. However, its tender shoots and leaves also contain valuable bioactive compounds, presenting an underexplored opportunity for functional food applications. Integrating these by-products into the food industry as functional additives or ingredients not only enhances resource utilization but also aligns with sustainable agricultural practices.

## 8 Conclusion

Polysaccharides from *P. cyrtonema* Hua possesses high medicinal and nutritional value due to their diverse array of chemical constituents. Traditional techniques like the ‘Nine-Steam-Nine-Bask’ method have proven effective in preserving the bioactivity of polysaccharides, although some studies suggest that the optimal number of cycles is four. The multifaceted health benefits of these polysaccharides present numerous potential applications in food and medicine. Obtaining homogeneous pure compounds and elucidating their structural properties are essential for expanding their applications. Further studies could also focus on bioavailability, pharmacokinetics, modifications, and comprehensive clinical trials. Addressing these research areas will enhance our understanding and application of PCH, ultimately contributing to improved health outcomes and sustainable practices across various industries.
